# Classification of Factors Effect on Sleep in Individuals with Down Syndrome

**DOI:** 10.3390/brainsci11111500

**Published:** 2021-11-12

**Authors:** Thai Duy Nguyen, Sébastien Baillieul, Michel Guinot, Stéphane Doutreleau, Véronique-Aurélie Bricout

**Affiliations:** University Grenoble Alpes, Inserm U1300, CHU Grenoble Alpes, HP2, Grenoble 38000, France; thainguyenduy@hotmail.com (T.D.N.); sbaillieul@chu-grenoble.fr (S.B.); MGuinot@chu-genoble.fr (M.G.); sdoutreleau@chu-grenoble.fr (S.D.)

**Keywords:** physical activity, sleep, Down syndrome, cluster analysis

## Abstract

Background: Sleep disordered breathing (SDB) is a frequent disorder with serious adverse health consequences in people with Down syndrome (DS). This study aims to evaluate and classify sleep and physical activity (PA) characteristics in adults with DS. Methods: Forty participants with DS wore an accelerometer for seven consecutive days to measure physical activity and sleep–wake patterns. The corresponding data were also obtained by survey and polysomnography. The apnea-hypopnea index (AHI) is calculated from the number of apneas and hypopneas per hour of sleep according to international guidelines. Results: Polysomnography showed SDB based on AHI in 95% of adults: 50% had severe SDB, 22.5% presented moderate and 22.5% mild SDB, predominantly in males. They had poor sleep quality (80.1%) and low PA levels. Using statistical classification methods, we observed three clusters with two opposite profiles. Clusters 2 and 3 showed low PA indices (daily steps: 5719 and 5310, respectively) and severe SDB (AHI = 33.6 and 55.5 events/h), high age and high gonial angle. Cluster 1 showed high PA (mean count of daily steps: 6908) and mild to moderate SDB (AHI = 16.8 events/h), low age and low gonial angle. Conclusions: Our findings suggest that there are different profiles (age, gender, obesity, gonial angle) that are associated with SDB in adults with DS. These results suggest that this may represent important factors to consider when planning health promotion and prevention.

## 1. Introduction

Down syndrome (DS) is the most frequent genetic disorder, associated with a wide spectrum of cognitive and physiological impairments, such as sleep disorders, obesity, hypotonia and also delays in motor and neurological development [1]. To improve life expectancy and quality of life, the medical follow-up of children and adults with DS is today well-framed by a set of recommendations [2,3]. Early nutritional advice, regular physical activities (PA) and targeted interventions from families of children with DS and/or from health professionals can help in reducing the risk or delaying the appearance of some of the DS-associated impairments, thus beneficially impacting their health. These nutritional and PA recommendations are an integral part of an overall healthy lifestyle, which sleep quality and quantity is fully a part of. However, patients with DS are prone to present sleep disordered breathing (SDB), such as obstructive sleep apnea syndrome (OSAS), frequently observed in up to 80–97% of patients with DS [4,5,6]. In adults with DS, OSA is usually more severe compared to controls because numerous factors are exacerbated by the clinical characteristics of this genetic syndrome, such as anatomical or endocrine factors which contribute to the physiopathology of SDB. Associated with difficult access to healthcare, these characteristics exacerbate the incidence of SDB in this population [7].

Polysomnograpy (PSG) is the gold standard examination to assess sleep quality and diagnose sleep-related disorders. OSA severity is assessed using the apnea-hypopnea index (AHI) with the following classification: no OSA: AHI < 5 events/h; Mild: 5 ≥ AHI < 15 events/h; Moderate: 15 ≥ AHI < 30 events/h; Severe: AHI ≥ 30 events/h. The consequences of the OSAS-related pathophysiological consequences and in particular sleep disruptions from infancy to adulthood may be particularly deleterious, affecting cognition, learning, behavior and inducing fatigue [4,8]. If PSG allows to efficiently assess sleep quality parameters, its costs, restricted availability and bothersome character in individuals with cognitive impairments may limit the possibility to conduct PSG in populations with DS [9].

One of the proposals retained to limit these deleterious consequences on health is regular PA. The benefits of exercise training in a population with DS have many positive effects on quality of life and global health outcomes [10,11]. Nevertheless, several authors reported the difficulties for persons with DS to engage in physical activity [12,13]. The reduced PA levels have a direct impact on sleep quality [14]. Their bidirectional relationships start being explored in youth and adults with DS, but their potential interactions require further understanding.

In the general population, PA is often assessed using self-report measures such as those obtained by questionnaires [15]. These measures are easily obtained and can provide information on the types of activities performed, but lack information regarding activity patterns (intensity and duration) throughout the day [16]. Moreover, obstacles can be observed when using self-report measures in persons with cognitive disabilities [17]. In this context, objective PA measures are required. Accelerometry assessments allow the collection of real-life data on PA characteristics (frequency, intensity and duration) [18] and sleep–wake patterns (SWP), such as total sleep time, sleep onset latency, wake up time, wake after sleep onset and sleep efficiency [19]. Thus, accelerometry has been used as an objective and non-invasive SWP assessment tool in populations with DS [20]. This study aimed to assess the PA and sleep characteristics (both subjective and objective) obtained by questionnaires, accelerometry and PSG in adults with DS. Statistical classification methods were applied to highlight the key elements affecting sleep parameters in this population.

## 2. Materials and Methods

This study was an observational prospective, monocentric study, conducted at the Grenoble Alpes University Hospital (France). It was approved by the National Ethics Committee Sud Méditerranée (France; n° 2017-AOI914-49) and was conducted according to the principles expressed in the Declaration of Helsinki. The clinical trial registration number is NCT 03445962. Each participant received oral and written information and signed a consent form.

### 2.1. Participants

Forty participants with a diagnosis of DS volunteered to participate in this study, including sixteen women and twenty-four men (29.7 ± 7.5 years; 18 to 46 years), with a moderate intellectual disability. They were recruited through local associations or employment support services. Participants had to be 18 or older and able to practice PA to be included in the study. All were of Caucasian origin, and lived with their parents. They were employed in an especially professional setting adapted for disability.

They were not included if they presented co-morbid medical (diabetes, leukemia, asthma or other respiratory infection) or psychiatric disorders, contraindications to physical activity (no cardiac insufficiency or cardiovascular disease) and severe osteo-articular pathology.

Included participants underwent PA and sleep parameters’ examinations subjectively assessed by questionnaires and objectively by accelerometry measurements and polysomnography.

Standardized lateral cephalograms were taken at the first visit, and the gonial angle was measured [21].

### 2.2. Questionnaires

Three questionnaires were completed by the participants and their parents, together, to assess sleep- and PA-related parameters.

The Pittsburgh Sleep Quality Index (PSQI) is the most globally used scale for sleep quality assessment. It consists of 19 items organized into 7 subscales: sleep quality, sleep latency, sleep duration, habitual sleep efficiency, sleep disturbances, use of sleeping medication and daytime dysfunction. The scores for each subcategory range between 0 and 3 with a total score of 21. The higher the score, the worse the sleep quality [22].

The Epworth Sleepiness Scale (ESS) is used to measure daytime sleepiness. It consists of 8 items scored from 0 to 3, which measure an individual’s propensity to fall asleep in everyday situations. A score between 10 and 16 represents moderate sleepiness, and a score > 16 represents severe sleepiness [23].

The Global Physical Activity Questionnaire (GPAQ) is a tool developed by the World Health Organization that collects information on PA levels in three situations: at work, during transport and leisure time. The questionnaire consists of sixteen questions on the time spent in vigorous or moderate PA in one of the three proposed situations. The score is then converted into metabolic equivalents (Mets) minutes per week. It can be calculated for the 3 activity (work, transport, leisure) and summed to get a total weekly PA score [24].

### 2.3. Actigraphy

Each participant wore an accelerometer (Actigraph GT9X^®^, Actigraph Corp, TSP diffusion, Poissy, France) for seven consecutive days, and one week before the PSG. This device was worn at the waist, on the right side. It can reliably measure SWP and PA parameters. Participants and/or their relatives (parent or educator) also completed daily diaries to identify periods in which participants were active, inactive or were lying down. Periods during which participants did not wear the accelerometer were not included in the analysis (shower or bath, swimming or other water activities). PA levels (sedentary, moderate and vigorous PA) and energy expenditure for PA were analyzed based on the Freedson algorithm adapted for adults [25]. The sleep-related variables used in this study were: total sleep time (min), sleep latency (defined as the difference between bedtime and sleep onset, with the information given by the algorithm of the device (binary coding: wake = 0, sleep = 1), in min), number of awakenings and sleep efficiency (calculated by the accelerometer as time in bed over the total sleep time, in %). PA variables were: daily step number (step), sedentary behavior duration (min) and moderate to vigorous PA duration (min).

### 2.4. Polysomnography (PSG)

PSG is considered as the gold standard for diagnosing sleep-related disorders, especially obstructive sleep apnea syndrome [26]. Overnight polysomnography was carried out at the hospital during one night before treatment for each subject if necessary. An overnight polysomnography (Deltamed, NATUS, France) was performed according to standard procedures [27]. The parameters monitored included electroencephalogram (Fz−A+, Cz−A+ and Pz−A+ of the international 10–20 Electrode Placement System), right and left electro-oculogram, submental electromyogram, tibial electromyogram, electrocardiogram and an overnight video monitoring.

Airflow was measured with nasal pressure prongs, together with the sum of oral and nasal thermistor signals. Respiratory effort was monitored using abdominal and thoracic bands. Oxygen saturation was measured using a pulse oximeter and oxygen desaturation index (ODI), and mean nocturnal SpO_2_ and percentage of recording time spent at a SpO_2_ < 90% were also calculated.

Sleep and respiratory events were scored manually according to the American Academy of Sleep Medicine (AASM) scoring manual v2.4 by trained sleep technologists, who were not blind from patient status. The apnea-hypopnea index (AHI) was calculated from the number of apneas and hypopneas per hour of sleep according to international guidelines [28]. An apnea was defined as the complete cessation or a reduction of at least 90% of airflow for at least 10 s and hypopnea as a reduction of at least 50% in the nasal pressure signal or a decrease of between 30% and 50% associated with either oxygen desaturation of at least 3% or an EEG arousal, both lasting for at least 10 s [29].

The sleep variables selected to be used in the analysis were total sleep period (including intra-sleep wake after sleep onset, min), total sleep time (min), sleep latency (min), number of awakenings, sleep efficiency (%) and apnea-hypopnea index (AHI), obtained exclusively by PSG examination.

### 2.5. Statistics

The data collected with the accelerometer were extracted using the software provided by the manufacturer (Actilife 6^®^ software Department, Pensacola, FL, USA). The average value of each variable (over seven days) was calculated. First, descriptive analyses were used to characterize the study population. Second, we aimed to identify the factors related to SDB using a classification model.

The descriptive analyses were performed using SPSS 20 and R software (version 4.0). Characterization of the population (*n* = 40) was carried out after checking the normality of the variables using the Shapiro–Wilk test and the homogeneity of the variances using Levene’s test. Paired *t*-tests were used to compare sleep- and PA-related variables collected using different methods (questionnaire, actigraphy, PSG). A linear regression was finally applied to investigate the relationship between sleep and PA variables. Results are presented as mean ± standard deviation (SD). The significance level was set at *p* < 0.05.

The classification model aimed to assess the impact of demographic factors and PA levels on SDB in adults with Down syndrome. The discriminant variable chosen was the AHI. Thirty-two variables were selected and divided into subgroups based on measurement methods: anthropometric, questionnaire, accelerometry and PSG parameters. A principal component analysis (PCA) and an agglomerative hierarchical cluster analysis (AHCA) were performed on data obtained for classification. The PCA was supplied with the normalized version of the original predictors. Here, normalization and centralization of the data by the feature scaling method were first applied [30]. In this part of our study, we had *n* = 40 observations (40 adults with DS) and 32 predictors, so a parsimonious model by the Elastic net algorithm was used to select the most relevant variables from a total of 32 variables [31]. Finally, the 6 predictive variables selected were: Age (a1), Gender (a2), BMI (a5), Gonial angle (a8), Daily step number (c5, accelerometry) and AHI (d6, PSG). This respected the required application rule for PCA: no more than 5 observations per prediction.

Lastly, a factor map was performed. This graphical representation offers a visualization of the different clusters at an individual level. Based on these observations, an average score was calculated in each cluster, using the following method for each variable: a score of 3 was assigned to the most favorable observation and a score of 1 to the least favorable. The value of the middle observation was 2. A subtotal was finally calculated for the sleep and PA variables. With these scores, it was possible to make a label to characterize each cluster.

## 3. Results

Descriptive demographic data, sleep-related and PA variables are presented in Table 1. Study participants were on average of short stature with body mass index indicating normal to overweight and large waist and neck circumferences. There was no significant difference between male and female participants in questionnaire scores and PA data obtained by activity measurements. They reported an overall good sleep quality (PSQI total score = 2.6) and no daytime sleepiness (Epworth total score = 4). These results contrast with the poor sleep efficiency observed during PSG (80.1%). We also observed lower total sleep time and sleep efficiency during PSG recordings in the male group compared to the female group (*p* < 0.05, Table 1).

Mean AHI for the entire group indicated severe SDB and was significantly higher in the male group (*p* < 0.05), and 95% of the study participants presented any degree of sleep apnea (AHI ≥ 5 events/h), with 50% of participants presenting sleep apnea (AHI ≥ 30 events/h), with a significant disparity between male and female participants (*p* < 0.05, Table 1). PA levels were low, especially the average daily steps number of 6231, and MVPA duration was also weak.

Regarding sleep and PA variables, significant statistical differences were found between the accelerometry method and the PSG method (Table 2). With the PSG method, total sleep time and sleep efficiency were lower than those evaluated with the accelerometer (391.7 min vs. 528.7 min; 80.1% vs. 92.0%, *p* < 0.01, Table 2). Sleep latency indices of sleep fragmentation such as number of awakenings assessed with the accelerometer were lower compared to PSG (4.8 min vs. 11.5 min; 10.7 vs. 34 events, *p* < 0.01, Table 2). 

The correlation test and linear regression analysis were performed to explore the relationship between AHI and all variables of interest. AHI was significantly and positively correlated with age (regression coefficient r = 0.33; *p* < 0.05) and gonial angle (regression coefficient r = 0.55; *p* < 0.01). 

**Figure 1:**
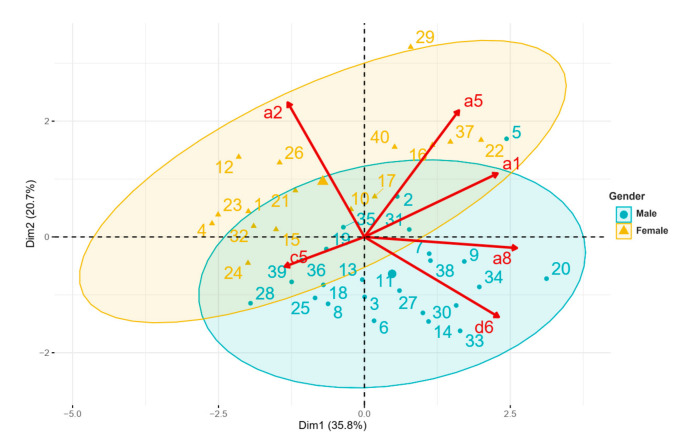
Principal component analysis biplot. Participants with DS are represented from 1 to 40. a1: age, a2: gender, a5: BMI (body mass index), a8: gonial angle, c5: daily steps number (accelerometry), d6: AHI (apnea-hypopnea index, PSG). On the biplot representation (Figure 1), a subject on the same side as a given variable obtained a high score for this variable. A low value for this variable was attributed to a subject on the opposite side. For example, a participant positioned near item d6 (case #33) presented a mean AHI of 76.69 events/h, and a participant on the opposite side (case #26) presented a mean AHI of 8.83 events/h.

Characteristics of SDB and related factors were explored by using PCA. Relationships were further explored using a clustering approach (AHCA). PCA results showed that 56.5% of the variance was explained by six significant variables grouped in the first two dimensions (35.8% and 20.7% respectively, eigenvalue ≥ 1), a satisfactory outcome. Regarding variable distribution (Figure 1), we observed that female participants with a low AHI score were grouped in the upper left quarter, while participants with a high AHI score where grouped in the lower right quarter. The lower left quarter regrouped young adults who had a normal BMI. Conversely, in the upper right quarter, the oldest participants with a high BMI were grouped. On this graphical representation, two participants were classified outside (#29, cluster 2) or near the border (#20, cluster 3) of the biplot representation. Participant #20 was characterized by a very high AHI score (99.9 events/h). He was 36 years old, and had a high BMI value (29), waist circumference (99 cm) and important sedentary behavior (525.43 min/24 h). Participant #29 was a woman of 36 years old, with a very high BMI value (35.6 cm) and waist circumference (100 cm).

The AHCA allowed us to classify the participants based on the demographic, sleep and PA variables. This analysis ranked each individual on a factor map, using different colors to indicate the membership of specific items from different clusters. 

**Figure 2:**
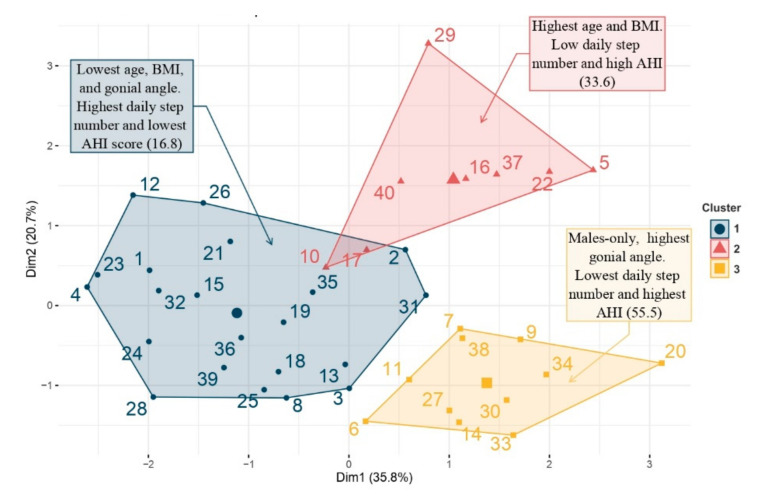
Factor map obtained by agglomerative hierarchical cluster analysis.

The representation with the factor map allowed us to specifically localize the position of one subject within its cluster and then compare it with neighboring clusters. Three clusters were obtained (Figure 2) composed respectively of 21 (Cluster 1, blue), 8 (Cluster 2, pink) and 11 (Cluster 3, yellow) participants. 

The 3 clusters are characterized in Table 3.

- Cluster 1 (blue) included twelve males and nine females with the lowest age, BMI and gonial angle values. They had the highest number of daily steps and lowest AHI score (16.8 events/h).
- Cluster 2 (red) included one male and seven females with the highest age and BMI value. They had a low number of daily steps and a high AHI score (33.6 events/h).
- Cluster 3 (yellow) included eleven males-only with the highest gonial angle value and higher age. They had the lowest number of daily steps and highest AHI score (55.5 events/h).

## 4. Discussion

This study aimed to investigate the PA and sleep characteristics obtained subjectively and objectively by questionnaires, accelerometry and polysomnography in adults with DS. This work presented a double originality, by providing multiple assessment methods and by focusing on adults with DS, for whom SDB diagnosis is sometimes overlooked. The results of our study showed that 95% of adults with DS have any degree of SDB. A major finding in this study is the discrepancies between PSG and the self-reported sleep questionnaires, which did not reflect the burden of sleep disturbances in this population.

Numerous studies have documented a high prevalence of OSA of up to 94% in people with DS [4,5,6], while this is found in only 13% of males and 6% of females in the general population [32]. OSA in the DS community tends to be more severe and can be associated with comorbidities such as obesity, cardiovascular diseases, dysautonomia and hypertension [33,34]. The higher severity in patients with DS may be related to anatomical, mechanical or endocrine factors as well as the difficulty to access healthcare in people with DS [7]. In this study, we classified sleep and PA characteristics to highlight the key elements that were related to AHI in adults with DS. The results confirmed significant associations between demographic characteristics such as age, gender, BMI and AHI in people with DS. These results are in line with previous studies [4,7,35].

People with DS of different ages present a higher prevalence of obesity [36]. High BMI is associated with an increased risk of severe OSA due to fat deposition at the neck level, which promotes narrowing of the upper airway diameter during sleep [7,35]. In a study on 16 adults with DS with a mean BMI of 31 kg/m^2^, Trois et al. reported a frequency of OSA of 88% [4]. Other studies had also shown a positive correlation between AHI and BMI in adults with DS [37]. 

The sleep assessment showed a high prevalence of SDB with an average AHI of 30.8 events/h (72.5% of participants with an AHI > 15; 50% of participants with an AHI ≥ 30 events/h). Among them, males presented more severe forms of SDB than females (average AHI 36.9 vs. 21.6 events/h). These results suggested that the tendency to have OSA was very common among obese people in the population with DS, especially males and participants with higher age.

With the comparison of the data obtained on the different sleep assessment modalities, we have found significant differences between the subjective method (questionnaires) and objective method (accelerometry, PSG) (Table 1 and Table 2). Questionnaire results related good sleep quality and low levels of daytime sleepiness (Table 1) while the sleep efficiency measured by PSG was poor, below the normal value of 85%. Many studies on the characteristics of sleep disorders in the population with DS are available, but are mainly focused on children rather than on adults. We observed that the total sleep time was shortened to an average of 6.5 h per night with a sleep efficiency of 80.1%, which may be related to sleep disruption associated with SDB (average AHI = 30.8 events/h). Regarding males, the situation was even worse, with an average total sleep time of 6.1 h per night and a sleep efficiency of 75.9% (Table 1), far below the recommended optimal sleep duration of 8 h or more per night in adults [38]. 

The second important outcome of this work was PA level evaluation and the assessment of its link with SDB. The benefits of regular physical exercise have been demonstrated in DS [8]. In our study, we found low levels of daily PA, especially the average number of daily steps, compared to the World Health Organization recommendations of 10,000 steps in adults [39]. According to these PA guidelines, adults should spend at least 30 min in MVPA per day at least 5 days a week [40]. In our study, time spent in MVPA was 26.4 min/day, associated with an important time spent in sedentary behavior (Table 2). However, data from cross-sectional studies have shown that a large proportion of aging subjects with DS engage in less and less PA, with a blunted response to exercise [41]. Many physical and mental barriers limit their participation in PA and increase sedentary behavior [13]. Several studies were reported on the reduction of the peak aerobic capacity (VO_2peak_), heart rate response (HR) and muscle strength in people with DS compared with people without disabilities [42,43]. They often suffered from cardiovascular, respiratory, musculoskeletal, intellectual disability and obesity [44], and these characteristics are factors in their lower engagement in PA.

Regular PA may normally be beneficial to promote sleep health and prevent or treat SDB, through weight loss and improvement in sleep latency and wake after sleep onset [44]. The decrease of sedentary behavior duration has demonstrated positive associations with weight loss [10,11]. More regular MVPA can also help to improve sleep quality [45].

PA limitation might contribute to sleep quality impairment. The negative effects of PA and motor dysfunction on increased SDB risk in populations with DS have been reported [7]. Obesity and physical fatigue modulated by sleep disruption are also well-described, and are associated with lower physical performance in people with DS [44]. In this study, cluster classification has highlighted a group of patients with several comorbidities: inactivity, obesity and SDB. It is by acting on these three elements that we will act on their health, but it is not by treating only the SDB problem that they will lose weight and increase their physical activity levels. PCA followed by AHCA allowed us to classify these participants into three groups, in which we observed significant characteristics of PA and other factors that affect SDB. Clusters 2 and 3 were characterized by a severe AHI associated with obesity, age and low PA level. Cluster 1 was characterized by young, normo-weighted subjects with a high PA level with no SDB. These results were consistent with those already reported by other authors who have shown strong associations between sleep disorders and age, weight status, gender and PA level in DS [44]. Participants in cluster 3 (only males) had the highest AHI, and were the most sedentary. The higher the body mass index, the less physical activity practiced, and the more sedentary behavior observed. These associations were similar to those reported in various studies about the effect of PA on sleep quality [44,45]. These authors have concluded that weight loss associated with regular PA has been recommended as one of the pertinent non-pharmacologic treatments, improving sleep quality as well as physical functions [46]. PA has a beneficial effect on mental health by reducing anxiety and depression, and increasing cognitive and adaptive abilities, thereby helping to improve sleep quality [47]. Moreover, it is also involved in thermoregulation, so the drop of temperature after PA helps to reduce sleep latency [48].

The originality of our study was also based on the use of three different methods of sleep assessment: questionnaires, accelerometry and PSG. Results indicated limitations with the methods even though mild to severe SDB was found in 95% of the sample. It was proven that the questionnaires were difficult to use and not suitable for the population with DS, who often have intellectual disabilities [49]. The accelerometry method seemed easy to use and provided more accurate information on SWP and PA in people with DS compared to the questionnaire [50]. PSG is the most reliable method for the assessment of sleep parameters and the diagnosis of SDB for the population with DS [51]. However, the differences observed between accelerometry and polysomnography may be due to limitations of monitoring equipment or failure of participants with DS to follow instructions during the PSG assessment because this examination was very constraining (many sensors and measuring devices), and as it was carried out in a hospital environment, it was anxiety-provoking [52]. 

Finally, it seems essential to make the earliest possible diagnosis of SDB, in order to then offer appropriate treatment. Patients with DS present some difficulties to adhere to standard SDB treatments, but if SDB remains untreated, deleterious consequences can occur [7]. Treatment of SDB in patients with DS can proceed along the same process as for all patients with surgery, lifestyle modifications or treatment by specific equipment [7]. The strong points of this study were the use of objective methods of sleep assessment (PSG) and PA evaluation coupled with questionnaires and the application of original statistical methods. Our study presents some limitations. To increase statistical power and confidence in PCA and AHCA results, higher recruitment of participants is necessary. Future studies should also consider adding a control group to increase the objectivity of the study results. 

## 5. Conclusions

Based on both subjective and objective assessments, our results indicated severe SDB in adults with DS, especially in males and older participants with higher age. We also found typical factors in participants with DS associated with a higher frequency of SDB (males, obese, older, high gonial angle and low PA). These results may be relevant to design tailored care pathways, to identify individuals at higher risk and to provide optimal care in this population.

Besides, this study highlighted that PA could represent a useful non-pharmacological method to be implemented in SDB care pathways for people with DS, but the nature and the level of this PA need to be properly determined taking into account with the physical and intellectual limitations encountered by patients with DS at an individual level. Future studies should investigate the mechanism and the magnitude of the impact of the PA interventions’ effect on sleep quality in people with DS.

## Figures and Tables

**Table 1:** Descriptive data.

		Total (*n* = 40)	Male (*n* = 24)	Female (*n* = 16)	*p*-Value
**Demographic**	Age (years)	29.7 ± 7.5	30.3 ± 6.2	28.8 ± 9.2	0.547
	Height (cm)	155.0 ± 8.0	160.5 ± 48.3	146.5 ± 47.8 *	0.0001
	Weight (kg)	60.0 ± 10.4	64.0 ± 9.0	53.9 ± 9.6 *	0.002
	BMI (kg/m^2^)	24.8 ± 3.9	24.7 ± 3.6	25.0 ± 4.4	0.765
	Waist circumference (cm)	87.0 ± 9.9	88.2 ± 8.2	85.3 ± 12.1	0.378
	Neck circumference (cm)	38.7 ± 3.5	40.7 ± 2.9	35.7 ± 2.1 *	0.0001
	Gonial angle	125.3 ± 8.4	126.9 ± 8.1	122.8 ± 8.6	0.138
	Gender (%)		60%	40%	
**PSG**	Total sleep time (min)	391.7 ± 88.1	366.1 ± 100.1	430.1 ± 46.6 *	0.010
	Sleep latency (min)	11.5 ± 9.5	12.6 ± 9.7	9.9 ± 8.3	0.375
	Number of awakenings	34.0 ± 15.3	33.8 ± 18.3	34.3 ± 9.6	0.926
	Sleep efficiency (%)	80.1 ± 15.6	75.9 ± 18.3	86.4 ± 6.9 *	0.015
	Total sleep period (min)	469.2 ± 55.8	458.5 ± 65.2	485.1 ± 33.5	0.142
	AHI (events/h)	30.8 ± 21.6	36.9 ± 23.8	21.6 ± 13.7 *	0.026
**Actigraphy**	Total sleep time (min)	528.7 ± 74.2	518.9 ± 78.4	543.3 ± 67.2	0.314
	Sleep latency (min)	4.8 ± 3.5	5.1 ± 3.9	4.2 ± 2.7	0.409
	Number of awakenings	10.7 ± 10.9	9.8 ± 9.0	12.1 ± 13.6	0.522
	Sleep efficiency (%)	92.0 ± 8.7	92.5 ± 7.6	91.3 ± 10.4	0.674
	Daily steps number	6231 ± 1746	6301± 1716	6125 ± 1841	0.759
	Sedentary behavior duration (min)	475.2 ± 89.6	469.5 ± 98.1	483.8 ± 77.5	0.627
	MVPA duration (min)	26.4 ± 19.2	29.4 ± 23.0	21.9 ± 10.2	0.233
**Question-naire**	PSQI total score	2.6 ± 1.9	2.5 ± 2.2	2.7 ± 1.5	0.681
	Epworth sleepiness scale total score	4.0 ± 2.4	4.1 ± 2.7	3.9 ± 1.8	0.727
	GPAQ total score	2572 ± 1912	2940 ± 2173	2019 ± 1311	0.103

Values are mean ± SD (*n* = 40). PSG (polysomnography), BMI (body mass index), AHI (apnea-hypopnea index), PSQI (Pittsburgh Sleep Quality Index), ESS (the Epworth Sleepiness Scale), GPAQ (the Global Physical Activity Questionnaire). * *p* < 0.05: significantly different between male and female.

**Table 2:** Characteristics of sleep- and AHI-based classification.

	Accelerometry	PSG	*p*-Value
Total sleep time (min)	528.7 ± 74.2	391.7 ± 88.1 **	0.001
Sleep latency (min)	4.8 ± 3.5	11.5 ± 9.1 **	0.001
Number of awakenings	10.7 ± 10.9	34.0 ± 15.3 **	0.001
Sleep efficiency (%)	92.0 ± 8.7	80.1 ± 15.6 **	0.001
	**Total (*n* = 40)**	**Male (*n* = 24)**	**Female (*n* = 16)**
No SDB (AHI < 5 events/h)	2	0	2
Mild (5 ≥ AHI < 15 events/h)	9	5	4
Moderate (15 ≥ AHI < 30 events/h)	9	5	4
Severe (AHI ≥ 30 events/h)	20	14	6 ^$^ (0.001)

Values are mean ± SD, *n* = 40. PSG (polysomnography), AHI (apnea-hypopnea index). ** *p* < 0.001: significantly different between accelerometry and PSG. ^$^: p < 0.05 significantly different between male and female.

**Table 3:** Average score in each cluster for all variables used in AHCA.

	Cluster 1 (*n* = 21)		Cluster 2 (*n* = 8)		Cluster 3 (*n* = 11)	
	Mean	Score	Mean	Score	Mean	Score
Gender *	57	2	12	3	100	1
Age	25.9	3	35.9	1	32.4	2
BMI	23.5	3	29.7	1	23.7	2
Gonial angle	120.2	3	130.5	2	131.0	1
Daily steps number	6908	3	5719	2	5310	1
AHI	16.8	3	33.60	2	55.5	1
Total score		17		11		8

BMI (body mass index), AHI (apnea-hypopnea index). *: Percent of male participants in each cluster. To calculate the scores: for example, the observation AHI, cluster 1 had a value of 10.5 (score = 3, Table 3) vs. a value of 38.1 for cluster 2 (score = 1, Table 3). This showed that cluster 1 has significantly less severe AHI than cluster 2.

## Data Availability

The data presented in this study are available on request from the corresponding author. The data are not publicly available due to privacy and ethical restrictions.
